# Micronutrient supplementation interventions in preconception and pregnant women at increased risk of developing pre-eclampsia: a systematic review and meta-analysis

**DOI:** 10.1038/s41430-022-01232-0

**Published:** 2022-11-09

**Authors:** Sowmiya Gunabalasingam, Daniele De Almeida Lima Slizys, Ola Quotah, Laura Magee, Sara L. White, Jessica Rigutto-Farebrother, Lucilla Poston, Kathryn V. Dalrymple, Angela C. Flynn

**Affiliations:** 1 Department of Women and Children’s Health, School of Life Course and Population Sciences, King’s College London, 10th Floor North Wing, St Thomas’ Hospital, Westminster Bridge Road, London, SE1 7EH UK; 2 Human Nutrition Laboratory, Institute of Food, Nutrition and Health, ETH Zurich, Zurich, Switzerland; 3 Department of Population Health Sciences, School of Life Course and Population Sciences, King’s College London, 4th floor Addison House, Guy’s Campus, London, SE1 1UL UK; 4 Department of Nutritional Sciences, School of Life Course and Population Sciences, King’s College London, Franklin-Wilkins Building, 150 Stamford Street, London, SE1 9NH UK

**Keywords:** Nutrition, Risk factors, Hypertension

## Abstract

**Background:**

Pre-eclampsia can lead to maternal and neonatal complications and is a common cause of maternal mortality worldwide. This review has examined the effect of micronutrient supplementation interventions in women identified as having a greater risk of developing pre-eclampsia.

**Methods:**

A systematic review was performed using the PRISMA guidelines. The electronic databases MEDLINE, EMBASE and the Cochrane Central Register of Controlled trials were searched for relevant literature and eligible studies identified according to a pre-specified criteria. A meta-analysis of randomised controlled trials (RCTs) was conducted to examine the effect of micronutrient supplementation on pre-eclampsia in high-risk women.

**Results:**

Twenty RCTs were identified and supplementation included vitamin C and E (*n* = 7), calcium (*n* = 5), vitamin D (*n* = 3), folic acid (*n* = 2), magnesium (*n* = 1) and multiple micronutrients (*n* = 2). Sample size and recruitment time point varied across studies and a variety of predictive factors were used to identify participants, with a previous history of pre-eclampsia being the most common. No studies utilised a validated prediction model. There was a reduction in pre-eclampsia with calcium (risk difference, −0.15 (−0.27, −0.03, I^2^ = 83.4%)), and vitamin D (risk difference, −0.09 (−0.17, −0.02, I^2^ = 0.0%)) supplementation.

**Conclusion:**

Our findings show a lower rate of pre-eclampsia with calcium and vitamin D, however, conclusions were limited by small sample sizes, methodological variability and heterogeneity between studies. Further higher quality, large-scale RCTs of calcium and vitamin D are warranted. Exploration of interventions at different time points before and during pregnancy as well as those which utilise prediction modelling methodology, would provide greater insight into the efficacy of micronutrient supplementation intervention in the prevention of pre-eclampsia in high-risk women.

## Introduction

Pre-eclampsia is a hypertensive disorder of pregnancy associated with a high risk of maternal, fetal and neonatal morbidity [1]. Pre-eclampsia has been defined as high blood pressure after 20 weeks’ gestation associated with one or more of the following: proteinuria, multisystemic maternal organ dysfunction or placental dysfunction [2, 3]. Pre-eclampsia affects around 2–8% of pregnancies globally, with approximately 10–15% of direct maternal deaths being attributed to pre-eclampsia and eclampsia [4].

The pathophysiology of pre-eclampsia is not fully understood and this disorder presents as a clinical syndrome with a wide spectrum [5]. Early onset pre-eclampsia is generally considered as a defect in placentation whilst late onset pre-eclampsia is more often attributed to a range of interacting factors including normal placental senescence and a genetic predisposition to cardiovascular and metabolic disease [5]. Poor placental function has repeatedly been associated with oxidative stress [6].

Several systematic reviews have assessed the effects of single and multiple micronutrients on the risk of developing pre-eclampsia. High dose calcium supplementation has been shown to be effective in reducing pre-eclampsia, particularly in women with low dietary calcium intake, but with limited evidence on the effects of low dose supplementation [7]. A recent umbrella review [8] reported that vitamin D supplementation reduced pre-eclampsia, while reporting limited or no effect of iron, folic acid supplementation or of the antioxidants, vitamin C and/or E. Despite magnesium being utilised in the treatment of pre-eclampsia and eclampsia, previous reviews have not been able to establish an effect of magnesium supplementation [9]. Similarly, many reviews [10, 11] report no established effect of zinc supplementation. There is increasing interest in the role of multiple micronutrient supplements (MMS) and their potential benefit for pregnant women, particularly in low-income countries where more than one micronutrient deficiency may co-exist. A meta-analysis of 28 RCTs [12] reported that despite evidence from observational cohort studies reporting a reduction in the risk of pre-eclampsia following MMS, there was a lack of effect from RCTs.

Interventions may be better targeted to women with more specific risk for adverse pregnancy outcomes. Several studies have used prediction modelling to identify those women more likely to develop pre-eclampsia [13–15]. An externally validated model from The Avon Longitudinal Study of Parents and Children cohort used routinely collected data to predict pre-eclampsia risk in a 12,996 women. The study combined maternal early pregnancy characteristics (including initial mean arterial pressure [MAP]) with repeatedly measured MAP collected from 20–36 weeks’ gestation. The authors found that blood pressure recorded from 28 weeks’ gestation improved the model’s identification of women who would go on to develop pre-eclampsia with an area under the curve of 0.81 and 0.83 in the validated cohort [13]. Other cohorts have frequently combined clinical risk factors with biomarkers and imaging techniques such as uterine artery Doppler ultrasound recorded in the first trimester [16]. Multivariable prediction models such as this have often demonstrated better performance with predicting early-onset pre-eclampsia [17]. The application of predictive modelling in the context of preventative micronutrient interventions in pre-eclampsia may offer insight into effectiveness of predictive factors and algorithms in clinical practice.

To the best of our knowledge, there has been no review of studies which have utilised prediction tools to stratify interventions intended to reduce pre-eclampsia. There is a need to evaluate interventions that utilise micronutrient supplementation in women who have been identified as high-risk for pre-eclampsia. Moreover, there is a paucity of data on the effects of pre-pregnancy micronutrient interventions on the development of pre-eclampsia. As healthcare aims to move towards primary prevention, it is important to assess supplementation interventions prior to pregnancy on the development of pre-eclampsia. Finally, few reviews report the effect of micronutrient supplementation in women with differing severity of pre-eclampsia (mild, severe and superimposed) who have been identified as high risk, which could allow more tailored and personalised preventative approaches in the future.

The overall aim of this review was to assess RCTs of micronutrient supplementation (single and multiple micronutrients) either pre-pregnancy and/or during pregnancy to prevent pre-eclampsia in women identified as high risk. An additional aim included examining the effect of intervention on different severities of pre-eclampsia in higher-risk women.

## Methods

This systematic review was registered in the PROSPERO database (CRD42021240941) and conducted according to the PRISMA guidelines [18].

### Inclusion and exclusion criteria

The PICOS (population, intervention, comparison, outcomes and study design) framework described in Table 1 was used to develop the inclusion and exclusion criteria. Studies were eligible if they met the following criteria: (1) RCTs evaluating single or multiple micronutrient supplementation before and/or during pregnancy compared with a control arm (no supplementation, placebo, dose difference or alternative micronutrient supplementation intervention) with a primary or secondary outcome of any classification of pre-eclampsia; (2) reproductive aged women between 18 and 50 years who intended to become pregnant or were pregnant at any gestation; (3) women identified as high risk of developing pre-eclampsia using a defined eligibility criteria at study entry. Studies meeting the following criteria were excluded: (1) observational and non-randomised studies; (2) abstracts, reviews, letters, comments and editorials; (3) women with existing pre-eclampsia; (4) women aged less than 18 years or more than 50 years; (5) studies not published in English.

**Table 1:** PICOS framework summary.

P – Population	Women between the ages of 18 to 50 who were planning to become pregnant or were pregnant at any gestation, and at high-risk for pre-eclampsia identified using a defined eligibility criteria at study entry
I – Intervention	Interventions included micronutrient supplementation, in isolation or as multiple micronutrients. This included, but was not limited to calcium, vitamin D, folic acid, iron, zinc and magnesium
C – Comparison	No supplementation, placebo, different dose or alternative micronutrient supplementation intervention
O – Outcome	The main outcome was the development of pre-eclampsia. Secondary outcomes included gestational hypertension, eclampsia, diastolic and systolic blood pressure, HELLP syndrome, premature rupture of membranes, placental abruption, preterm birth, low birthweight, birth weight centile, small for gestational age (SGA), caesarean section, miscarriage, Apgar scores and maternal death
S – Study type	Randomised controlled trials

### Primary and secondary outcomes

The primary outcome of this review was the development of pre-eclampsia of any classification including mild, severe and superimposed pre-eclampsia, defined by any diagnostic criteria ranging from the use of systolic (SBP) and diastolic blood pressures (DBP), urinary protein measurements and other relevant clinical indicators such as liver enzymes and platelet count. Trials that reported definitions for severe pre-eclampsia generally defined this with the same diagnostic criteria, however with higher thresholds for blood pressure and urinary protein measurement. The secondary outcomes included gestational hypertension, eclampsia, diastolic and systolic blood pressure, HELLP syndrome, premature rupture of membranes, placental abruption, preterm birth, low birthweight, birth weight centile, small for gestational age (SGA), caesarean section, miscarriage, Apgar scores and maternal death, of which 7 of these secondary outcomes have been identified as part of the recommended core outcome set for pre-eclampsia for future studies [19]. Data were extracted on secondary outcomes from studies when available.

### Literature search and study selection

The electronic databases MEDLINE, EMBASE and the Cochrane Central Register of Controlled trials were searched by two reviewers (SG, DDALS) on 14th April 2021. Search strategies are shown in Supplementary Information 1. Results of the search strategy were imported into EndNote for removal of duplicates and the remaining articles were imported into Rayyan for title and abstract screening [20]. If eligibility could not be determined by the title and abstract, full-text articles were screened. Any disagreement was resolved by a third reviewer (ACF). Additional studies were examined for eligibility through hand searching of reference lists.

### Data extraction

Two reviewers (SG, DDALS) carried out the data extraction in duplicate and any disagreements were resolved by discussion or by consultation with another author to achieve a consensus opinion (ACF and KVD). A data extraction template was developed which included: title, authors, publication data, trial periods, study design, country of study, aim of study, sample size, characteristics of participants, inclusion and exclusion criteria, period of intervention (preconception and/or pregnancy), type and dose of intervention and clinical outcomes.

### Data synthesis

The interventions and outcomes were assessed for suitability for data pooling to perform a meta-analysis. The analysis focused on assessing the effect of micronutrient interventions (single or multiple micronutrients) on the development of pre-eclampsia, the primary outcome, in high-risk populations. Micronutrient interventions included calcium, vitamin D, vitamin C and E, folic acid, magnesium and MMS in women defined as high risk of developing any classification of pre-eclampsia including severe pre-eclampsia, as previously defined. Summaries of exposure effect for each intervention were provided using a risk difference, calculated using Stata, version 16. The risk differences were calculated using a random effects model and the I^2^ statistic was used to assess heterogeneity amongst studies, with a threshold of >50% indicating significant heterogeneity. When meta-analysis was not possible due to too few studies, a narrative synthesis was performed.

### Risk of bias (quality) assessment

The Cochrane Risk of Bias tool for randomised trials (RoB 2) [21] was used to assess the quality of each study included. The domains assessed include randomisation selection (selection bias), allocation concealment (selection bias), follow-up of participant from recruitment to termination of study and dropout (attrition bias) and other potential sources of bias. Disagreement between reviewers was resolved by discussion, with the overall risk of each study being deemed as either “low risk of bias,” “some concerns” or “high risk of bias.”

## Results

**Fig. 1:**
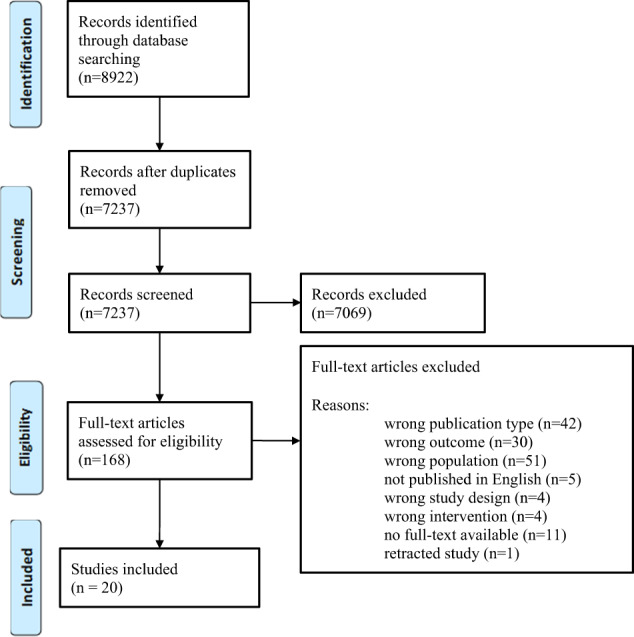
PRISMA Flow Diagram. Flow diagram showing the study selection process.

The electronic database search resulted in 8922 articles. Following removal of duplicates, a total of 7237 articles were screened for eligibility using titles and abstracts. Full-text screening was conducted on 168 articles. Major reasons for exclusion included: publication type (e.g. commentary articles or protocols of clinical trials), ineligible outcome (e.g. studies that did not include pre-eclampsia in the results), ineligible population (e.g. low risk women, adolescents), studies which were not published in English, ineligible study design (e.g. review and observational studies), incorrect type of intervention (e.g. pharmacological intervention) and retraction of trials. Twenty articles [22–41] met the inclusion criteria and were included in this review. The study identification and selection process are summarised in Fig. 1.

### Characteristics of included studies

This review included RCTs published between 1991 to 2021. A summary of these RCTs is shown in Table 2. The characteristics of studies including women identified as high-risk for pre-eclampsia are shown in Table 3. Six studies [23, 24, 29, 30, 32, 38] were conducted in Iran, three studies [31, 33, 39] in the United States, two studies [25, 40] in the United Kingdom, two studies [26, 34] in Brazil, one study in each of Colombia [27], India [28], Mexico [35] and China [36]. Three studies were multicentre and were conducted in South Africa, Zimbabwe and Argentina [22]; India, Peru, South America and Vietnam [37]; Argentina, Australia, Canada, Jamaica and the United Kingdom [41]. Fifteen studies were carried out in low- and middle-income countries [22–24, 26–30, 32, 34–38, 41]. The lowest sample size was 50 [28] and the largest sample size 2464 [41]. The studies aimed to assess the efficacy of either the supplementation of calcium, vitamin D, vitamin C in combination with vitamin E, magnesium, folic acid or MMS in reducing the incidence of pre-eclampsia in women classified at high-risk of pre-eclampsia at study entry. Of the 20 studies, two targeted women before pregnancy [18, 32]. The earliest time a pregnancy intervention was introduced was at 8 weeks’ gestation [41] with most of the studies continuing the micronutrient supplementation until delivery.

**Table 2:** Summary of included studies.

Reference	Publication date	Period of intervention	Country of study	Micronutrient supplementation intervention	Screening for pre-eclampsia	Gestational age during intervention
Hofmeyr et al.	2019	Prepregnancy into pregnancy	South Africa, Zimbabwe, Argentina	Calcium	Previous pre-eclampsia	From prepregnancy to 20 weeks’ gestation
Baba Dizavandy et al.	1998	Pregnancy	Iran	Calcium	Positive roll-over test	From 28–32 weeks gestation to delivery
Herrera et al.	1998	Pregnancy	Colombia	Calcium and linoleic acid	Positive roll-over test and high mean arterial pressure	From 28–32 weeks’ gestation to delivery
Niromanesh et al.	2001	Pregnancy	Iran	Calcium	Positive roll-over test and at least one risk factor for pre-eclampsia	Until delivery
Sanchez-Ramos et al.	1994	Pregnancy	USA	Calcium	Positive angiotensin sensitivity test	Not reported
Behjat et al.	2017	Pregnancy	Iran	Vitamin D3	Previous pre-eclampsia	Until 36 weeks’ gestation
Karamali et al.	2015	Pregnancy	Iran	Vitamin D3	Uterine artery doppler	From 20 weeks’ to 32 weeks’ gestation
Samimi et al.	2015	Pregnancy	Iran	Vitamin D3 and calcium	Laboratory tests including free ß-human chorionic gonadotrophin, inhibin α dimeric, unconjugated oestriol and maternal serum α-foetoprotein and haemodynamic assessment of uterine artery Doppler waveform at 16–20 weeks of gestation	From 20 weeks’ to 32 weeks’ gestation
Chappell et al.	1999	Pregnancy	UK	Vitamin C and vitamin E	Uterine artery doppler and previous pre-eclampsia	From 16–22 weeks’ gestation (depending on prior history of pre-eclampsia) to delivery
Poston et al.	2006	Pregnancy	UK and Holland	Vitamin C and vitamin E	One or more risk factors for pre-eclampsia (previous pre-eclampsia, delivery <37 weeks, diagnosis of HELLP in previous pregnancy, essential hypertension requiring medication currently or previously, maternal diastolic blood pressure of ≥90 mmHg before 20 weeks’ gestation in the current pregnancy, type 1 or type 2 diabetes requiring insulin or oral hypoglycaemic therapy before the pregnancy, antiphospholipid syndrome, chronic renal disease, multiple pregnancy, abnormal uterine artery doppler waveform, primiparity with BMI at first antenatal appointment of ≥30)	From 14–21 weeks’ gestation to delivery
Spinatto et al.	2007	Pregnancy	Brazil	Vitamin C and vitamin E	Previous pre-eclampsia in most recent pregnancy that progressed beyond 20 weeks’ gestation	From 12–19 weeks’ gestation to delivery
Kalpdev et al.	2010	Pregnancy	India	Vitamin C and vitamin E	Essential hypertension	From 16–22 weeks’ gestation to delivery
Beazley et al.	2005	Pregnancy	USA	Vitamin C and vitamin E	Previous pre-eclampsia, chronic hypertension, insulin-requiring diabetes or multiple gestation	From 14–20 weeks’ gestation
Vadillo-Ortega et al.	2011	Pregnancy	Mexico	Vitamin C and vitamin E	Previous pre-eclampsia or pre-eclampsia in first-degree relative	From 14–32 weeks’ gestation to delivery
Villar et al.	2009	Pregnancy	India, Peru, South Africa, Vietnam	Vitamin C and vitamin E	High risk for pre-eclampsia (chronic hypertension, renal disease, pre-eclampsia-eclampsia in the pregnancy preceding the index pregnancy requiring delivery before 37 weeks’ gestation, HELLP syndrome in any previous pregnancy, pregestational diabetes, primiparous with a BMI ≥ 30, history of preterm delivery, abnormal uterine artery Doppler waveforms and women with antiphospholipid syndrome)	14–22 weeks’ gestation to delivery
Zheng et al.	2020	Prepregnancy into pregnancy	China	Folic acid	Previous pre-eclampsia	Preconception to delivery
Wen et al.	2018	Pregnancy	Argentina, Australia, Canada, Jamaica and UK	Folic acid	At least one risk factor for pre-eclampsia (pre-existing hypertension, prepregnancy type 1 or type 2 diabetes, twin pregnancy, previous pre-eclampsia or BMI ≥ 35)	From 8–16 weeks’ gestation to delivery
De Araujo et al.	2020	Pregnancy	Brazil	Magnesium	≥1 risk factor for preterm birth or adverse perinatal outcomes in a prior pregnancy (i.e. preterm delivery <37 weeks, still birth at 20^1/7^ weeks, placental abruption, pre-eclampsia or eclampsia, a live-born infant with SGA birthweight <3rd percentile or liveborn infant with birthweight <2500 g) or in current pregnancy (i.e. nulliparity, chronic hypertension, type 1 or 2 diabetes mellitus, maternal age >35 years, pre-pregnancy BMI > 30 or smoking)	From 12–20 weeks’ gestation to delivery
Azami et al.	2017	Pregnancy	Iran	MMS	At least one risk factor for pre-eclampsia (including chronic vascular disease, hydatidiform mole, multiparity, diabetes mellitus, thyroid disease, chronic hypertension, nulliparity, history of pre-eclampsia, maternal age >35 years, kidney disease, collagen vascular disease, antiphospholipid antibody syndrome, family history of pre-eclampsia, history of thrombophilia and BMI > 25)	From 20 weeks’ gestation to delivery
Parrish et al.	2013	Pregnancy	US	MMS	Previous pre-eclampsia, prior eclampsia, history of chronic hypertension, diabetes mellitus, connective tissue disease or inherited/acquired thrombophilia	Any time up to 12 weeks’ gestation until delivery

**Table 3:** Characteristics of included studies.

Reference	Publication date	Period of intervention	Study design	Sample size	Study period	Country of study	Study aim
Hofmeyr et al.	2019	Prepregnancy into pregnancy	Multicentre, parallel arm, double-blind, randomised, placebo-controlled trial	*N* = 1355	2011–2016	South Africa, Zimbabwe, Argentina	To test whether calcium supplementation before and in early pregnancy (up to 20 weeks’ gestation) prevents the development of pre-eclampsia
Baba Dizavandy et al.	1998	Pregnancy	Double-blind randomised trial	*N* = 143	Not reported	Iran	To determine the effect of calcium supplementation in the incidence of hypertensive disorders of pregnancy (gestational hypertension and pre-eclampsia) in nulliparous and high risk women
Herrera et al.	1998	Pregnancy	Randomised double-blind placebo-controlled trial	*N* = 89	1995–1996	Colombia	To determine the effect of low doses of linoleic acid and calcium on prostaglandin (PG) levels and the prevention of pre-eclampsia
Niromanesh et al.	2001	Pregnancy	Double-blind placebo randomised controlled trial	*N* = 30	Not reported	Iran	To study the effect of calcium supplementation on reduction of pre-eclampsia in Iranian women at high risk of pre-eclampsia
Sanchez-Ramos et al.	1994	Pregnancy	Randomised, double-blind, placebo-controlled clinical trial	*N* = 67	Not reported	USA	To evaluate the efficacy of oral supplemental calcium in reducing the incidence of pregnancy-induced hypertension (gestational hypertension or pre-eclampsia) in angiotensin sensitive nulliparas
Behjat et al.	2017	Pregnancy	Randomised double-blinded controlled clinical trial	*N* = 142	Not reported	Iran	To evaluate if vitamin D supplementation prevents pre-eclampsia in women with history of pre-eclampsia
Karamali et al.	2015	Pregnancy	Randomised double-blind placebo-controlled trial	*N* = 60	2014	Iran	To assess the beneficial effects of high-dose (cholecalciferol) vitamin D supplementation on metabolic profiles and pregnancy outcomes among pregnant women at risk for pre-eclampsia
Samimi et al.	2015	Pregnancy	Prospective, double-blind, placebo-controlled randomised trial	*N* = 60	2014–2015	Iran	To examine the effects of vitamin D plus calcium administration on metabolic profiles and pregnancy outcomes among women at risk for pre-eclampsia
Chappell et al.	1999	Pregnancy	Randomised controlled trial	*N* = 283	Not reported	UK	To assess the effect of supplementation with vitamin C and E in women at increased risk of the disorder on plasma markers of vascular endothelial activation and placental insufficiency and the occurrence of pre-eclampsia
Poston et al.	2006	Pregnancy	Randomised controlled trial	*N* = 2404	2003–2005	UK and Holland	To assess whether supplementation with vitamin C and vitamin E prevents pre-eclampsia in women at increased risk
Spinatto et al.	2007	Pregnancy	Randomised controlled trial	*N* = 739	2003–2006	Brazil	To evaluate whether antioxidant supplementation will reduce the incidence of pre-eclampsia among patients at increased risk
Kalpdev et al.	2010	Pregnancy	Randomised controlled trial	*N* = 50	2005–2007	India	To investigate whether vitamin C and vitamin E prophylaxis will reduce the incidence of superimposed pre-eclampsia in chronic hypertensive women
Beazley et al.	2005	Pregnancy	Double-blind randomised controlled trial	*N* = 109	Not reported	USA	To determine the effect of supplemental antioxidant vitamins C and E on the rate of pre-eclampsia in high-risk pregnant women
Vadillo-Ortega et al.	2011	Pregnancy	Randomised controlled trial	*N* = 628	2001–2005	Mexico	To test the hypothesis that deficiency in L-arginine and antioxidant supplementation would reduce the development of pre-eclampsia in a population at high risk
Villar et al.	2009	Pregnancy	Multi-centre, randomised, double-blind controlled trial	*N* = 1365	2004–2006	India, Peru, South Africa, Vietnam	To determine if vitamin C and E supplementation in high-risk pregnant women with low nutritional status reduces pre-eclampsia
Zheng et al.	2020	Prepregnancy into pregnancy	Randomised controlled trial	*N* = 1576	Not reported	China	To investigate whether supplementation with high doses of folic acid would reduce the subsequent development of pre-eclampsia and its adverse outcomes
Wen et al.	2018	Pregnancy	Randomised, phase III, double-blind, international multi-centre clinical trial	*N* = 2464	2011–2015	Argentina, Australia, Canada, Jamaica and UK	To determine the efficacy of high dose folic acid supplementation for prevention of pre-eclampsia in women with at least one risk factor: pre-existing hypertension, prepregnancy diabetes (type 1 or 2), twin pregnancy, pre-eclampsia in a previous pregnancy, or body mass index ≥35
De Araujo et al.	2020	Pregnancy	Randomised double-blinded controlled clinical trial	*N* = 911	2014–2017	Brazil	To evaluate magnesium citrate to prevent adverse perinatal and maternal outcomes among women at higher risk
Azami et al.	2017	Pregnancy	Randomised controlled trial	*N* = 100	Not reported	Iran	To investigate the effect of multimineral-vitamin D supplements (calcium, magnesium, zinc and vitamin D) and vitamins (C + E) in the prevention of pre-eclampsia
Parrish et al.	2013	Pregnancy	Randomised, placebo-controlled double-blind trial	*N* = 684	2004–2011	US	To evaluate if phytonutrient supplementation initiated in the first trimester of pregnancy and continued throughout gestation prevents pre-eclampsia

### Predictive factors of pre-eclampsia

The studies used a variety of strategies to identify women at risk of developing pre-eclampsia. The number of variables used ranged from one to 15 and included a range of factors from maternal history to clinical biomarkers (Table 4). Thirteen studies [22, 24–26, 31, 34–41] considered a history of pre-eclampsia in a prior pregnancy, with two [35, 38] of these also including family history of pre-eclampsia. Seven studies [26, 31, 37–41] required participants to have at least one risk factor of pre-eclampsia such as chronic hypertension, type 1 or 2 diabetes mellitus, multiple gestation, or history of preterm birth. Five studies [25, 29, 32, 37, 40] used uterine artery doppler waveforms to select participants, either in isolation or within a combination of other maternal characteristics. Three studies [23, 27, 30] included a positive rollover test, while only one study [33] utilised a positive angiotensin sensitivity test. This test was performed in women at 24–28 weeks’ gestation, by infusing increasing doses of angiotensin II every 5 min until the normal cut-off value (12 ng/kg/min) was reached or the DBP increased to 20 mmHg above the baseline before reaching this cut-off value (the effective pressor dose). If a participant’s effective pressor dose was achieved with a rate of less than 12 ng/kg/min, they were deemed to have a positive angiotensin sensitivity test and were then randomised. Three studies [25, 27, 30] required participants to have a combination of predictive factors, for example, both an abnormal two-stage uterine-artery doppler analysis and a previous history of pre-eclampsia [25]. None reported using a validated prediction model to identify women at high-risk of developing pre-eclampsia.

**Table 4:** Screening at study entry and micronutrient supplementation interventions in included studies.

Reference	Screening for pre-eclampsia	Intervention	Gestational age during intervention
Hofmeyr et al.	Women with previous pre-eclampsia and were intending to become pregnant again	I = 500 mg of calcium carbonate daily from prepregnancy until 20 weeks’ gestation C = Placebo	From prepregnancy to 20 weeks’ gestation
Baba Dizavandy et al.	Nulliparous women with singleton pregnancies, 24 weeks of gestation, blood pressure <140/90 mmHg, a positive roll-over test and hypocalcuria at 28–32 weeks of pregnancy	I = 2000 mg of calcium gluconate daily C = Placebo	From 28–32 weeks gestation to delivery
Herrera et al.	First pregnancy, gestation between 28–32 weeks, biopsychosocial risk score of 3 or more, positive roll-over test and high mean arterial pressure	I = 50 mg of linoleic acid and 600 mg of calcium daily C = Placebo	From 28–32 weeks’ gestation to delivery
Niromanesh et al.	High-risk for pre-eclampsia (identified as having positive results on rollover test and at least one risk factor for pre-eclampsia), gestational age between 28–32 weeks, blood pressure <140/90 mmHg	I = 2000 mg of calcium daily C = Placebo	Until delivery
Sanchez-Ramos et al.	Normotensive nulliparas at 20–24 weeks’ gestation at increased risk of developing pregnancy-induced hypertension (through positive angiotensin sensitivity test)	I = 2000 mg of calcium daily C = Placebo	Not reported
Behjat et al.	Pre-eclampsia in previous pregnancy, serum 25-hydroxy vitamin D levels >25 ng/ml	I = 50000 IU of vitamin D3 every 2 weeks C = Placebo	Until 36 weeks’ gestation
Karamali et al.	Pregnant women primigravida, age of 18–40 and risk of pre-eclampsia (identified through uterine artery Doppler)	I = 50000 IU of vitamin D3 every 14 days C = Placebo	From 20 weeks’ to 32 weeks’ gestation
Samimi et al.	Primigravida women, aged 18–40 years old, at risk for pre-eclampsia (indicated by laboratory tests including free ß-human chorionic gonadotrophin, inhibin α dimeric, unconjugated oestriol and maternal serum α-foetoprotein and haemodynamic assessment of uterine artery Doppler waveform at 16–20 weeks of gestation)	I = 50000IU of vitamin D3 every 2 weeks and 1000 mg calcium daily C = Placebo	From 20 weeks’ to 32 weeks’ gestation
Chappell et al.	Women with abnormal two-stage uterine-artery doppler analysis weeks and previous history of pre-eclampsia	I = 1000 mg of vitamin C and 400 IU of vitamin E daily C = Placebo	From 16–22 weeks’ gestation (depending on prior history of pre-eclampsia) to delivery
Poston et al.	Gestational age 14–21 weeks 6 days and one or more risk factors for pre-eclampsia including pre-eclampsia in the pregnancy preceding the index pregnancy, requiring delivery before 37 completed weeks’ gestation, diagnosis of HELLP syndrome in any previous pregnancy at any stage of gestation, essential hypertension requiring medication currently or previously, maternal diastolic blood pressure of ≥90 mmHg before 20 weeks’ gestation in the current pregnancy, type 1 or type 2 diabetes requiring insulin or oral hypoglycaemic therapy before the pregnancy, antiphospholipid syndrome, chronic renal disease, multiple pregnancy, abnormal uterine artery doppler waveform, primiparity with BMI at first antenatal appointment of ≥30	I = 1000 mg of vitamin C and 400IU vitamin E daily C = Placebo	From 14–21 weeks’ gestation to delivery
Spinatto et al.	Pregnant women between 12 and 19 6/7 weeks gestation, non-proteinuric chronic hypertension or with a prior history of preeclampsia in their most recent pregnancy that progressed beyond 20 weeks gestation	I = 1000 mg of vitamin C and 400IU of vitamin E daily C = Placebo	From 12–19 weeks’ gestation to delivery
Kalpdev et al.	Essential hypertension, singleton pregnancy, gestational age 16 to 22 weeks	I = 1000 mg of vitamin C and 400IU of vitamin E daily C = No supplementation	From 16–22 weeks’ gestation to delivery
Beazley et al.	Pregnancy at 14–20 weeks and 6 days with a history of previous pre-eclampsia, chronic hypertension, insulin-requiring diabetes or multiple gestation	I = 1000 mg of vitamin C and 400IU of vitamin E daily C = Placebo	From 14–20 weeks’ gestation
Vadillo-Ortega et al.	Increased risk of pre-eclampsia (history of pre-eclampsia or pre-eclampsia in a first degree relative)	I = 6.6 g of L-arginine + 500 mg of vitamin C + 400IU of vitamin E daily I2 = 500 mg of vitamin C + 400IU of vitamin E daily C = Placebo	From 14–32 weeks’ gestation to delivery
Villar et al.	Pregnant women 14–22 weeks of gestation and with high risk for pre-eclampsia (chronic hypertension, renal disease, pre-eclampsia-eclampsia in the pregnancy preceding the index pregnancy requiring delivery before 37 weeks’ gestation, HELLP syndrome in any previous pregnancy, pregestational diabetes, primiparous with a BMI ≥ 30, history of preterm delivery, abnormal uterine artery Doppler waveforms and women with antiphospholipid syndrome)	I = 1000 mg of vitamin C and 400IU of vitamin E daily C = Placebo	14–22 weeks’ gestation to delivery
Zheng et al.	Previous pre-eclampsia, planning pregnancy, aged over 18 years, daily folic acid intake before randomization <1.1 mg	C = Low dose folic acid (0.4 mg) daily I = High dose folic acid (4 mg) daily	Preconception to delivery
Wen et al.	Pregnant women between 8–16 completed weeks of gestation and at least one risk factor for pre-eclampsia including pre-existing hypertension, prepregnancy diabetes (type 1 or 2), twin pregnancy, pre-eclampsia in a previous pregnancy or BMI ≥ 35	I = 4 mg of folic acid daily C = Placebo	From 8–16 weeks’ gestation to delivery
De Araujo et al.	Women aged 18 to 45 years, 12 to 20 weeks of gestation, singleton pregnancy and ≥1 risk factor for preterm birth or adverse perinatal outcomes in a prior pregnancy (i.e. preterm delivery <37 weeks, still birth at 20^1/7^ weeks, placental abruption, pre-eclampsia or eclampsia, a live-born infant with SGA birthweight <3rd percentile or liveborn infant with birthweight <2500 g) or in current pregnancy (i.e. nulliparity, chronic hypertension, type 1 or 2 diabetes mellitus, maternal age >35 years, pre-pregnancy BMI > 30 or smoking)	I = 300 mg of magnesium citrate daily C = Placebo	From 12–20 weeks’ gestation to delivery
Azami et al.	Women with at least one risk factor for pre-eclampsia (including chronic vascular disease, hydatidiform mole, multiparity, diabetes mellitus, thyroid disease, chronic hypertension, nulliparity, history of pre-eclampsia, maternal age >35 years, kidney disease, collagen vascular disease, antiphospholipid antibody syndrome, family history of pre-eclampsia, history of thrombophilia and BMI > 25)	I1 = Ferrous sulfate tablet + one multimineral vitamin D tablet containing 800 mg of calcium, 8 mg of zinc and 400IU of vitamin D3 daily I2 = Ferrous sulfate tablet + 250 mg of vitamin C and 55 mg of vitamin E daily C = Ferrous sulfate tablet daily	From 20 weeks’ gestation to delivery
Parrish et al.	For low risk group: nulliparous or multiparous women, singleton gestation and no evidence of systemic vascular disease For high risk group: multiparous patients with singleton gestation and a prior history of preeclampsia (prior eclampsia, prior mild or severe preeclampsia, prior HELLP) or nulliparous/multiparous patients with singleton gestation with a documented history of chronic hypertension, diabetes mellitus, connective tissue disease or inherited/acquired thrombophilia	I = Phytonutrients (7.5 mg beta-carotene, 234 mg vitamin C, 30 mg vitamin E, 420 mg folate and 60 mg calcium - mix through a concentrate of blended fruit and vegetable juice powder) taken twice daily until delivery C = Placebo	Any time up to 12 weeks’ gestation until delivery

### Micronutrient interventions

The type of intervention varied between studies (Table 4). The majority evaluated the effect of single micronutrients including calcium [22, 23, 27, 30, 33], combined vitamin C and E [25, 28, 34, 35, 37, 39, 40], vitamin D [24, 29, 32], folic acid [36, 41] and magnesium [26]. Two studies evaluated the effect of MMS which included multimineral-vitamin D supplements (calcium, magnesium, zinc and vitamin D) compared to vitamins C and E [38] and phytonutrient supplementation [31].

Seventeen studies compared the micronutrient supplementation intervention to a placebo, whilst one study compared the intervention to standard clinic protocol [28]. One study compared the effects of different dosages of micronutrient supplementation [36] another study made comparisons between MMS and vitamin C and E supplementation [38].

### Calcium supplementation

**Fig. 2:**
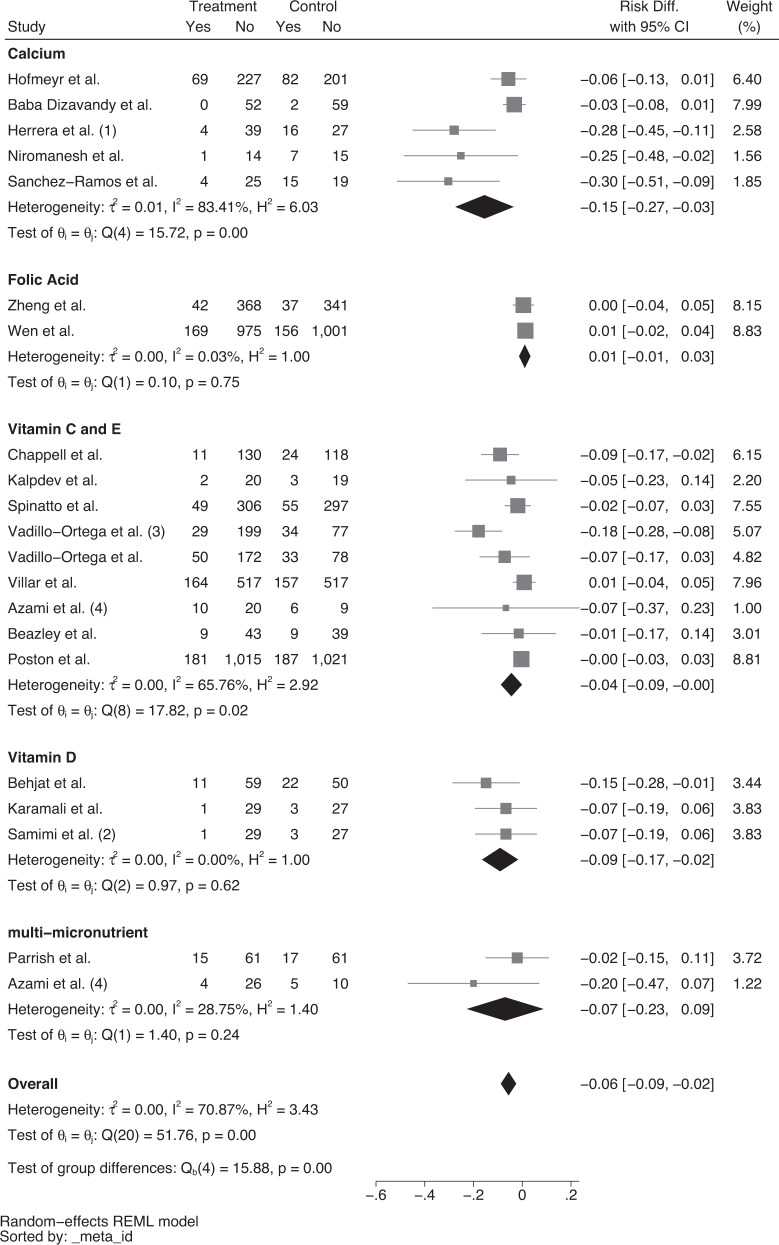
Forest plot of risk difference (95% CI) for overall pre-eclampsia, stratified by supplementation. Forest plot assessing risk of overall pre-eclampsia with calcium, folic acid, vitamin C and E, vitamin D or multi-micronutrient supplementation. Key: (1) includes linoleic acid (2) includes calcium (3) includes L-arginine (4) all participants given ferrous sulfate tablets.

**Fig. 3:**
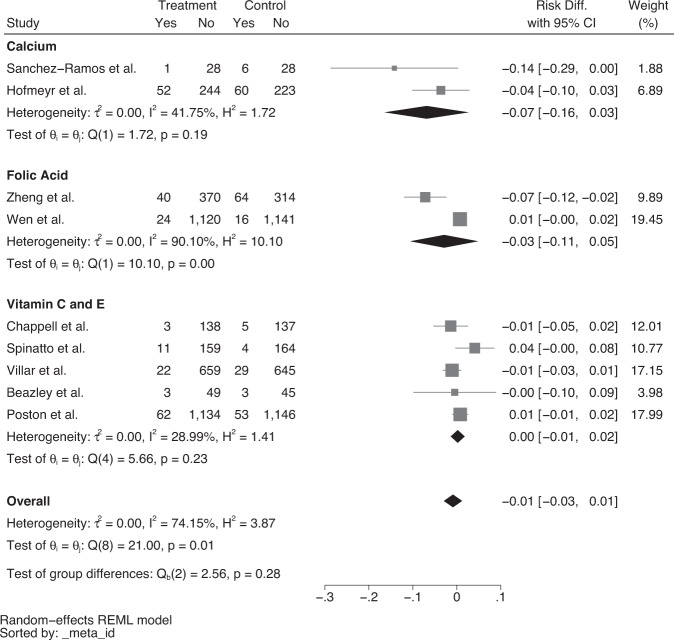
Forest plot of risk difference (95% CI) for severe pre-eclampsia only, stratified by supplementation. Forest plot assessing risk of severe pre-eclampsia with calcium, folic acid or vitamin C and E supplementation.

Three calcium interventions used a high dose between 1500 to 2000 mg daily [23, 30, 33]. Two studies [22, 27] used a lower dose of 500 and 600 mg calcium, respectively. Hofmeyr et al. started 500 mg of calcium supplementation before pregnancy until 20 weeks’ gestation whilst Herrera et al. used a 600 mg calcium supplement with 50 mg of linoleic acid between 28 to 32 weeks’ gestation until delivery [22, 27]. Similarly, Babadizavandy et al. utilised 2000 mg of calcium supplementation between 28 to 32 weeks’ gestation until delivery [23]. Both Niromanesh et al. and Sanchez-Ramos et al. also used 2000 mg of daily calcium supplementation. However, the period of the intervention was unclear [30, 33]. Of the 2 trials that reported data on compliance, Sanchez-Ramos et al. had a compliance rate of 91.1% while Hofmeyr et al. trial reported that approximately 50% of participants took at least 80% of their expected tablets. Random effects meta-analysis of five studies showed a lower rate of pre-eclampsia with calcium supplementation (risk difference = −0.15, 95% CI = −0.27 to −0.03), with significant heterogeneity among the studies (I^2^ = 83.41%, Fig. 2), however, there was no reduction in severe pre-eclampsia (Fig. 3).

### Vitamin D supplementation interventions

All three studies used a dose of 50,000 IU of vitamin D every 2 weeks, with 2 using this intervention in isolation [24, 29] and the third in combination with 1000 mg of calcium [32], with a reported compliance rate of 90–100% across all three trials. All 3 studies were conducted in the pregnancy period until 32 to 36 weeks’ gestation. There was a lower risk of pre-eclampsia with vitamin D supplementation in comparison to no supplementation (risk difference = −0.09, 95% CI = −0.17 to −0.02, I^2^ = 0.00%, Fig. 2), however there was no effect on severe pre-eclampsia (Fig. 3).

### Vitamin C and E supplementation interventions

All 7 studies [25, 28, 34, 35, 37, 39, 40] used vitamin C and E together in combination with 6 studies using a dose of 1000 mg vitamin C and 400 IU vitamin E [25, 28, 34, 37, 39, 40]. The remaining study used a dose of 500 mg vitamin C and 400 IU vitamin E with or without L-arginine [35]. The majority of studies started the intervention in the second trimester of pregnancy, ranging from 14 to 24 weeks’ gestation, whilst Spinnato et al. initiated the intervention at the end of the first trimester [34]. Five trials reported a moderate to high level of compliance [25, 34, 35, 37, 40].

Random effects meta-analysis showed that the risk difference for pre-eclampsia was 4% for vitamin C and E, (risk difference = −0.04, 95% CI = −0.09 to 0.00) however, there was significant heterogeneity between the studies (I^2^ = 65.76%, Fig. 2). There was no effect on the rate of severe pre-eclampsia. Poston et al. reported no significant effect of vitamin C and E supplementation in the prevention of pre-eclampsia, however, infants born with a low birthweight were higher in the intervention group (I = 387 vs. C = 335, *p* = 0.023, RR = 1.15, 95% CI = 1.02–1.30).

### Folic acid supplementation interventions

One study evaluated the effect of high dose folic acid (4 mg daily) compared to low dose folic acid (0.4 mg daily) from before pregnancy until delivery [36], whilst one study assessed the effect of the daily supplementation of 4 mg of folic acid from 8 to 16 weeks’ gestation until delivery [41]. Zheng et al. assessed homocysteine plasma levels and reported that levels were significantly lower in the high dose group which had a compliance rate of 74.1% compared to 73% in the low dose group. Among the high dose group, the incidence of pre-eclampsia was reduced when compliance was greater than 80% compared to 50%. Overall, there was no effect of folic acid supplementation on pre-eclampsia (Fig. 2) or severe pre-eclampsia (Fig. 3).

### Magnesium supplementation interventions

De Araújo et al. assessed the effect of 300 mg of daily magnesium supplementation compared to a placebo from 12 to 20 weeks’ gestation until delivery and showed no significant impact on pre-eclampsia or severe pre-eclampsia [26]. Although adherence was defined in this trial, compliance rates were not reported.

### MMS interventions

The two studies [31, 38] that used multiple micronutrients differed according to composition. Azami et al. compared three groups of pregnant women who received a daily ferrous sulphate tablet with a multimineral-vitamin D tablet consisting of 800 mg calcium, 9 mg zinc and 400 IU vitamin D (Group A), a daily ferrous sulphate tablet with vitamin C and vitamin E (Group B) or a daily ferrous sulphate tablet alone (Group C) [38], while Parrish et al. assessed the effect of a combination of phytonutrients including 7.5 mg of beta-carotene, 234 mg of Vitamin C, 30 mg of vitamin E, 420 mg of folate and 60 mg of calcium, taken twice daily until delivery, compared to placebo [31]. There were no data reported on compliance.

Pooled estimate of the 2 studies showed that MMS was not associated with a reduction in overall pre-eclampsia (Fig. 2). Parrish et al. also showed no reduction in the rate of severe pre-eclampsia [31].

### Diagnostic criteria for pre-eclampsia

The studies diagnosed and classified pre-eclampsia using primarily SBP and DBP, urinary protein measurements and other clinical indicators such as liver enzymes and platelet count (Table 5).

**Table 5:** Diagnostic criteria and outcomes of included studies.

Reference	Diagnostic criteria for pre-eclampsia	Pre-eclampsia	Classifications of pre-eclampsia	Other outcomes
Hofmeyr et al.	Gestational hypertension (DBP > 90 mmHg on two occasions 4 h apart, or >110 mmHg once, or SBP > 140 mmHg on two occasions 4 h apart, or >160 mmHg once after 20 weeks’ gestation) and gestational proteinuria (2 r more on urine dipstick, or >300 mg/24 h or 500 mg/L or urinary protein:creatinine ratio >0.034 g/mmol after 20 weeks’ gestation) as diagnosed by the attending clinicians. Severe pre-eclampsia (proteinuria + severe DBP [>110 mmHg] or systolic [>160 mmHg] hypertension)	I = 69/296 (23%) vs C = 82/283 (29%), [RR = 0·80 (95% CI 0·61–1·06)], *p* = 0.121	I = 52/296 (18%) vs C = 60/283 (21%), [RR = 0.83 (95% CI 0.59–1.16)], *p* = 0.268	Not significant
Baba Dizavandy et al.	Having both gestational hypertension (SBP increase ≥30 mmHg and DBP increase ≥15 mmHg on two occasions at least 6 h apart, or SBP ≥ 140 mmHg and DBP ≥ 90 mmHg after 24 weeks’ gestation in absence of proteinuria) and proteinuria (>0.3 g/L urine on at least two separate random urine specimens more than 6 h apart after 24 weeks’ gestation)	I = 0% vs C = 3.3%	Not reported	Gestational hypertension: I = 11.4% vs C – 31.2%, *p* < 0.01 Pregnancy-induced hypertension: I = 11.4% vs C = 35.6%, *p* < 0.01 Serum calcium levels vs. SBP in C group: *r* = −0.28, *p* = 0.02
Herrera et al.	Development of hypertension from 20 weeks’ gestation (≥140/90 mmHg where there is an increase of ≥20 mmHg in DBP compared to previous levels during pregnancy on at least two occasions 6 or more hours apart) and significant 24-hour proteinuria (>0.3 g/L) in the absence of a urinary tract infection	I = 4 (9.3%) vs C = 16 (37.2%), [RR = 0.25], *p* = 0.002	Not reported	DBP: I = 74.6 ± 11.2 mmHg vs C = 81.9 ± 11.9 mmHg, *p* = 0.001 Gestational age at birth: I = 39.3 ± 1.4 weeks vs C = 38.2 ± 2.3 weeks, *p* = 0.03 Caesarean: I = 10 (23.3%) vs C = 19 (44.2%), *p* = 0.04 Birth weight: I = 3180 ± 340 g vs C = 3056 ± 475 g, *p* = 0.03 PGE2 levels after 30 days: I = + 106% vs C = −33%, *p* = 0.02
Niromanesh et al.	SBP of ≥140 mmHg (an increase of 30 mmHg) and DBP ≥ 90 mmHg (an increase of 15 mmHg) on two occasions at an interval of 4–6 h with proteinuria (1 + proteinuria on random sampling of urine, measured as sulfosalicylic acid)	I = 1/15 vs C = 7/15, *p* = 0.014	Not reported	Time of onset of pre-eclampsia: I = 37 weeks vs C = 34 weeks, *p* < 0.05 Time of onset of hypertension: I = 37 ± 2 weeks vs C = 34 ± 1.9 weeks, *p* < 0.040 Duration of pregnancy: I = 39.5 ± 0.8 weeks vs C = 37.7 ± 2.5 weeks, *p* < 0.05 Infant birth weight: I = 3316 ± 308 g vs C = 2764 ± 761 g, *p* < 0.05
Sanchez-Ramos et al.	BP ≥ 140/90 mmHg measured twice at 4–6 h apart and significant proteinuria (≥1+ on dipstick or at least 300 mg/2 h)	I = 13.8% vs C = 44.1%, *p* = 0.01, [RR = 0.37, (95% CI 0.15–0.92)]	Mild pre-eclampsia: I = 3/29 vs C = 9/34 Severe pre-eclampsia: I = 1/29 vs C = 6/34	Pregnancy-induced hypertension: I = 31.0% vs C = 64.7%, *p* = 0.01 [RR = 0.46, 95% CI 0.25–0.86]
Behjat et al.	BP ≥ 140/90 mmHg in sitting position with proteinuria of ≥1+ on urine dipstick	I = 11 (15.7%) vs C = 22 (30.6%), *p* = 0.036 Risk in the control group was 1.94 times higher (95% CI 1.02–3.71)	Not reported	Not reported
Karamali et al.	Not reported	I = 3.3% vs C = 10.0%, *p* = 0.30	Not reported	Serum vitamin D levels: I = 17.92 ± 2.88 ng/ml vs C = 0.27 ± 3.19 ng/ml, *p* = 0.001 Increase in insulin levels: I = 1.08 ± 6.90 µIU/ml vs C = 9.57 ± 10.32 µIU/ml, *p* < 0.001)
Samimi et al.	Not reported	I = 3.3% C = 10.0%, *p* = 0.30	Not reported	Change in DBP: I = −2.0 mmHg, SD 6.6 mmHg vs C = + 3.7 mmHg, SD 6.3 mmHg, *p* = 0.001 Change in SBP: I = −3.8 mmHg, SD 5.8 mmHg vs C = + 1.7 mmHg, SD = 8.7 mmHg, *p* = 0.006 Mean serum 25(OH) D concentration: I = 8.2 ng/mL, SD = 7.7 ng/mL vs C = + 0.1 ng/mL, SD = 3.2 ng/mL, *p* < 0.001 Change in FPG: I = −5.7 mg/dL, SD = 5.5 mg/dL vs C = −0.6 mg/dL, SD = 12.6 mg/dL, *p* = 0.04 Change in insulin: I = −0.28 µIU/ml, SD = 6.0 µIU/ml vs C = + 7.7 µIU/ml, SD = 9.0 µIU/ml, *p* < 0.001 Change in HOMA-IR: I = −0.8, SD = 1.3 vs C = + 1.6, SD = 2.2, *p* < 0.001 Change in HOMA-B: I = −8.2, SD = 25.8 vs C = + 32.6, SD = 41.3, *p* < 0.001 Change in QUICKI score: I = + 0.02, SD = 0.02 vs C = −0.02, SD – 0.02, *p* < 0.001 Change in Serum HDL cholesterol: I = + 4.6 mg/dL, SD = 8.3 mg/dL vs C = −2.9 mg/dL, SD = 7.7 mg/dL, *p* = 0.001 Change in plasma GSH concentration: I = + 23.4 µm, SD = 124.0 µm vs. C = −94.8 µm, SD = 130.2 µm, *p* = 0.001
Chappell et al.	Two recordings of DBP ≥ 90 mmHg at least 4 h apart (for severe pre-eclampsia, two recordings of DBP ≥ 110 mmHg at least 4 h apart or one reading of 120 mmHg) and proteinuria (excretion of 300 mg or more in 24 h or two readings of ≥2+ on midstream urine dipstick or catheter urine if 24-hour collection is not available) Superimposed pre-eclampsia was defined as the new development of proteinuria in women with chronic hypertension	ITT I = 11/141 (8%) vs C = 24/142 (17%) [Adj OR = 0.39 (95% CI 0.17–0.90)], *p* = 0.02	Mild pre-eclampsia: ITT I = 5/141 vs C = 13/142 Severe pre-eclampsia: ITT I = 3/141 vs C = 5/142 Superimposed pre-eclampsia: ITT I = 3/141 vs C = 6/142	PAI-1:PAI-2 ratio: 21% reduction in I (85% CI 4–35), *p* 0.015
Poston et al.	Gestational hypertension (two or more readings of DBP ≥ 90 mmHg taken at least 4 h and up to 168 h apart, occurring after 20 weeks’ gestation or up to 48 h in the early postnatal period, excluding labour) or severe gestational hypertension (same as above however with a DBP ≥ 110 mmHg on two or more occasions or a single reading of ≥120 mmHg), and proteinuria (excretion of 300 mg/24 h protein or two readings of ≥2+ on urine dipstick analysis) or severe proteinuria (excretion of ≥5000 mg/24 h)	I = 181 (15%) vs C = 187 (16%) [RR = 0·97, 95% CI 0·80–1·17], *p* = 0.754	Severe pre-eclampsia: I = 62 (5%) vs C = 53 (4%) [RR = 1.17, 95% CI 0.82–1.68]	Gestational hypertension: I = 84 (7%) vs. C = 55 (5%), [RR = 1.53, 95% CI 1.10–2.13] Low birth weight: I = 387 (28%) vs C = 335 (24%), *p* = 0.023 [RR = 1.15, 95% CI 1.02–1.30] Low birth weight in women with diabetes: I = 20% {*n* = 19) vs C = 10% (*n* = 6), risk ratio = 3.26 95% CI 1.36–7.84
Spinatto et al.	SBP ≥ 140 mmHg and DBP ≥ 90 mmHg or proteinuria (either 300 mg/24 hours or ≥2+ by dipstick on two or more occasions 4 h apart) Severe pre-eclampsia defined as severe hypertension (if ≥ 2 SBP values obtained 4 or more hours apart were 160 mmHg or if ≥2 DBP values were 110 mmHg) and proteinuria; urinary protein excretion 5 g/day with any degree of hypertension; hypertension complicated by pulmonary oedema or a low platelet count (<100,000/mL); or haemolysis, an elevated serum aspartate aminotransferase concentration (>70 units/L), and a low platelet count (HELLP syndrome). Superimposed pre-eclampsia was defined as hypertension + proteinuria (either 300 mg/24 h or ≥2+ by dipstick) in chronically hypertensive patients	ITT I = 49/355 (13.8%) vs C = 55/352 (15.6%), [Adj RR = 0.87, (95.42% CI 0.61–1.25)], *p* = 0.43	Severe pre-eclampsia in those without chronic hypertension: I = 11/170 (6.5%) vs C = 4/168 (2.4%), *p* = 0.11, [OR = 2.78, 95% CI 0.79–12.62]	Premature rupture of membranes: I = 10.6% vs C = 5.5%, *p* = 0.015 [RR = 1.89, 95% CI 1.11–3.23]
Kalpdev et al.	Superimposed pre-eclampsia was defined as the new onset proteinuria of ≥300 mg/24 h in hypertensive women with no proteinuria before 20 weeks’ gestation or a sudden increase in proteinuria or BP or platelet count of <100,000 mm^3^ in women with hypertension and proteinuria before 20 weeks’ gestation	N/A	Superimposed pre-eclampsia: I = 8% vs C = 12%, *p* = 1.000	FRAP levels: I = 1168.95 ± 191.32 µM vs C = 835.93 ± 162.35 µM, *p* = 0.022
Beazley et al.	Not reported	I = 17.3% vs C = 18.8% [RR = 0.92, 95% CI 0.4–2.13]	Severe pre-eclampsia: I = 3/52 vs 3/48 Total cases of mild pre-eclampsia: 5 Total cases of superimposed pre-eclampsia: 7	Not significant
Vadillo-Ortega et al.	Hypertension (SBP ≥ 140 mmHg, DBP ≥ 90 mmHg, or both) and proteinuria (>300 mg/24 h) after 20 weeks’ gestation in women known to be previously normotensive Severe pre-eclampsia defined as proteinuria >2.0 g/24 h, BP ≥ 160/110 mmHg or both	Incidence of pre-eclampsia and eclampsia together: L-arginine + antioxidant vitamins (I) vs placebo (C) = lower incidence in I [χ2 = 19.41, *p* < 0.001, ARR 0.17, 0.12–0.21] No differences between antioxidants (I2) group vs placebo (C), *p* = 0.052	Not reported	Preterm delivery: I = ARR = 0.53 [95% CI 0.33–0.84, χ2: *p* = 0.003], I2 = ARR = 0.44 [95% CI 0.28–0.70, χ2: *p* < 0.001] Side-effects: Significantly more side-effects in I than C: nausea (*p* = 0.019), symptoms of dyspepsia (*p* = 0.04), dizziness (*p* = 0.039), palpitations (*p* = 0.019) and headache (*p* = 0.09) 1-minute Apgar score: I vs C ARR = 0.44, 95% CI% 0.24–0.64, *p* = 0.000 I2 vs C ARR = 0.25, 95% CI = 0.04–0.47, *p* = 0.015 I vs I2 ARR = 0.18, 95% CI 0.01 = 0.36, *p* = 0.04 5-minute Apgar score: I vs C ARR = 0.21, 95% CI 0.10–0.33, *p* = 0.000
Villar et al.	De novo hypertension (≥2 readings of DBP ≥ 90 mmHg, taken 4 h apart or more, but <160 h apart, and occurring after 20 weeks’ gestation) and new-onset proteinuria (excretion of ≥300 mg/24 h or two readings of >1+ on dipstick if 24-h collection is not available) Severe pre-eclampsia defined as SBP ≥ 160 mmHg and/or DBP ≥ 110 mmHg on two occasions, at least 4 h but not more than 168 h apart, or if the first measurement was immediately followed by treatment with an antihypertensive, either of these scenarios being associated with proteinuria.	I = 24.1% vs C = 23.3% [RR = 1.0, 95% CI 0.9–1.3]	Severe pre-eclampsia: I = 3.2% vs C = 4.3% [RR = 0.8, 95% CI 0.4–1.3]	Not significant
Zheng et al.	BP ≥ 140/90 mmHg and proteinuria Severe pre-eclampsia diagnostic criteria not reported	Overall pre-eclampsia: Low dose group = 37/378 (9.8%) vs High dose group = 42/410 (10.2%) [RR = 0.96, 95% CI 0.76–1.19)], *p* = 0.684	Severe pre-eclampsia: Low dose group = 64/378 (16.9%) vs High dose group = 40/410 (9.8%) [RR = 1.69. 95% CI 0.55–4.80], *p* = 0.011	Severe gestational hypertension: Low dose group = Low dose group = 126/378 (33.3%) vs high dose group = 89/410 (21.7%), *p* = 0.021 [RR = 1.54, 95% CI = 0.67–3.70] Early onset of pre-eclampsia (<32 weeks): Low dose group = 73/378 (19.3%) vs high dose group = 52/4410 (12.7%), *p* = 0.039 [RR = 1.52, 95% CI 0.53–4.20] Apgar score <7 at 5 min: Low dose group = 5/378 (1.3%) vs high dose group = 3/410 (0.7%), *p* = 0.013 [RR = 1.85, 95% CI 0.44–4.32]
Wen et al.	DBP ≥ 90 mmHg on two occasions 4 or more hours apart and proteinuria (≥2+ on dipstick, or urinary protein ≥300 mg/24 h, or random protein:creatinine ratio ≥30 mg protein/mmol) in women at ≥20 weeks’ gestation, or diagnosis of HELLP syndrome or superimposed pre-eclampsia (history of pre-existing hypertension diagnosed before pregnancy or before 20 weeks’ gestation with new proteinuria) Severe pre-eclampsia diagnostic criteria not reported	I = 169/1144 (14.8%) vs C = 156/1157 (13.5%) [RR = 1.10, 95% CI 0.90–1.34] *p* = 0.37	Severe pre-eclampsia: I = 21/1144 (1.84%) vs C = 16/1156 (1.38%) [RR = 1.52, 95% CI 0.81–2.84], *p* = 0.19	Not significant
De Araujo et al.	Elevated SBP > 140 mmHg or DBP > 90 mmHg, with ≥2+ proteinuria on urine dipstick, and/or HELLP syndrome	I = 24 (5.9%) vs C = 20 (4.7%), [unadjusted RR = 1.24 (0.70 to 2.22)], [Adj OR = 1.25 (0.68 to 2.31)]	Not reported	Placental abruption: I = 9 (2.2%) vs C = 21 (5.0%), unadjusted RR = 0.44 (0.21–0.96), Adj OR = 0.43 (0.20–0.95)
Azami et al.	BP ≥ 140/90 after 20 weeks’ gestation and proteinuria ≥300 mg/24 hours or 1+ on urine dipstick	Group A (I) = 13.3% vs Group C (C) = 36.7%, *p* = 0.03 Group B (I) = 33.3% vs Group C (C) = 36.7%, *p* = 0.50 Group A vs Group B, *p* = 0.063	Not reported	Neonatal complications (unspecified): Lower in Group A (*p* = 0.01)
Parrish et al.	Not reported	High and low risk group: I = 15.9% vs C = 16.3%, *p* = 0.93 Low risk group: I = 10.7% vs C = 8.8%, *p* = 0.73, [RR = 1.22, 95% CI 0.40–3.77] High risk group: I = 19.7% vs C = 21.9%, *p* = 0.75, [RR = 0.91, 95% CI 0.49–1.68]	Mild pre-eclampsia: Low risk group I = 8.9% vs C = 8.8%, *p* = 0.98 [RR = 1.02, 95% CI 0.31–3.32]; high risk group I = 2.6% vs C = 1.3%, *p* = 0.99 [RR = 1.03, 95% CI 0.07–16.1] Severe pre-eclampsia: Low risk group I = 0% vs C = 0%; high risk group I = 5.3% vs C = 3.9%, *p* = 0.67 [RR = 1.37, 95% CI 0.32–5.91] Superimposed pre-eclampsia: Low risk group I = 0% vs C = 0%; high risk group I = 11.8% vs C = 16.7%, *p* = 0.40 [RR = 0.71, 95% CI 0.32–1.56]	Not significant

*RR* relative risk, *OR* odds ratio, *ARR* absolute risk reduction, *I* intervention, *C* control, *ITT* intention to treat, *Adj* adjusted, *SD* standard deviation, *BP* blood pressure, *DBP* diastolic blood pressure, *SBP* systolic blood pressure’

### Quality of included studies

The quality of the studies included in this review is shown in Supplementary Information 2. In total, 12 trials [22, 24, 25, 27, 29, 32–35, 37, 40, 41] were assessed as ‘low risk of bias,’ seven trials [23, 28, 30, 31, 36, 38, 39] as “high risk of bias” and one [26] was classified as “some concerns.” The main source of bias across the studies was either lack of information available on compliance and adherence in the study or non-adherence to the micronutrient supplementation intervention.

## Discussion

This review aimed to evaluate the effect of micronutrient supplementation interventions on the development of pre-eclampsia in women identified as high risk. Our findings showed a lower rate of pre-eclampsia with calcium and vitamin D supplementation. There was no effect of micronutrient supplementation on severe pre-eclampsia. The review was limited by studies not adequately powered to detect a difference in pre-eclampsia and heterogeneity between studies was high.

Calcium supplementation has been previously reported to reduce the risk of pre-eclampsia [7] with the greatest effects observed in women with low calcium diets, however doses of calcium varied significantly across the trials, ranging from 500 mg to 2000 mg. The mechanism is largely unknown; however it is suggested that calcium lowers blood pressure [42] and may also reduce activation of the vascular endothelium [43]. Our findings support the use of calcium, in women with low dietary intake, to reduce pre-eclampsia in high-risk women, however, the studies had a small sample size and were under powered for the outcome [22]. The variance amongst these trials, ranging from different diagnostic criteria to the differences in sample size, highlights the need for more well-powered and larger-scale trials to establish when and how calcium supplementation can be the most beneficial to women identified as high risk of developing pre-eclampsia.

The vitamin D supplementation trials showed a slight reduction in pre-eclampsia, however, from studies with a small sample size. Previous reviews have reported on the lack of consistent evidence of benefit of vitamin D supplementation and its role in the prevention of pre-eclampsia, adding that inconsistencies in reporting the timing and duration of the intervention have not yet been adequately addressed [44]. Our findings in high-risk women support this, with some studies lacking clarity in reporting the intervention components [24] and time of gestation during the intervention, highlighting the need for further studies in higher risk women and better reporting in clinical trials.

There was a slightly lower rate of pre-eclampsia with antioxidants (vitamin C and E), however the confidence interval included zero and adverse outcomes such as low birthweight [40] were higher in the intervention arm. Previous reviews have reported no benefit of vitamin C and E supplementation on pre-eclampsia in ‘all-risk’ women, however there might be a protective effect in low- and middle-income countries [8]. This review provides some evidence that vitamin C and E supplementation may reduce pre-eclampsia in higher-risk women by 4%, including in low- and middle-income countries [35], however secondary outcomes were unfavourable, including a higher instance of SGA infants in the intervention arm. Oxidative stress is known to play a pivotal role in the manifestation of conditions such as pre-eclampsia [45], and alternative methods of reversing oxidative stress or poor antioxidant status may be worth investigating further in higher risk women.

This review found no evidence of benefit of folic acid supplementation on the development of pre-eclampsia. A recent 2018 review of observational studies [46] reported that folic acid was associated with lower risk of developing pre-eclampsia in pregnancy, perhaps being most effective in combination with multivitamins. However, RCTs evaluating the effect of folic acid on the development of pre-eclampsia are scarce, with conflicting data about the optimal dosages and whether it is best in isolation or combination with other micronutrients [46] and suggests a need for further research in women identified as high risk. Appropriately designed RCTs which encompass both dosage comparison of folic acid and stratify participants by high vs low risk are needed to elucidate the role of folic acid in the prevention of pre-eclampsia.

A limited number of studies addressed the impact of magnesium. Additionally, few studies assessed effects of MMS supplements with considerable variation in the combination of micronutrients used in each trial. A 2019 review reported improvements in preterm birth, low birth weight and SGA when supplemented with MMS together with iron and folic acid [47], however the effect of MMS on pre-eclampsia is still unclear. Higher-quality studies are needed to evaluate the potential benefits of MMS on the prevention of pre-eclampsia with a focus on finding the most effective combination of micronutrients. We did not retrieve any studies assessing the efficacy of zinc in this review.

Specific classifications of pre-eclampsia were reported in 11 studies [22, 25, 28, 31, 33, 34, 36, 37, 39–41], including mild, severe and superimposed pre-eclampsia. There was no effect of micronutrient supplementation interventions on severe pre-eclampsia. Tailoring micronutrients and their dosages to predicted severity of pre-eclampsia may be more beneficial than more generalised preventative approaches, particularly in this stratified population as opposed to all-risk women. With a lack of reviews evaluating the effect of micronutrient supplementation interventions on different classifications of pre-eclampsia, future studies on pre-eclampsia and its various severities, along with more exploration of the associated predictive factors, could inform primary prevention strategies to improve maternal healthcare.

The studies in this review used a wide variety of predictive factors at study entry. Of the eight studies [24, 25, 27, 30, 33, 35, 36, 38] with positive findings, five [24, 25, 35, 36, 38] used a history of pre-eclampsia to identify high-risk women whilst two studies used a positive roll-over test in addition to another predictive factor [27, 30], with the remaining study using a positive angiotensin sensitivity test [33]. A history of pre-eclampsia was the most common factor used to select participants at study entry, with very few studies using a combination of more than one risk factor. Predictive factors have shown to be most effective through a combination of using maternal characteristics, biomarkers and other variables such as the uterine artery doppler [17]. For example, a new first-trimester screening algorithm combining MAP, uterine artery doppler and circulating levels of placental growth factor (PIGF) to predict preterm pre-eclampsia, has a true positive rate of 82%, almost double the rate of detection via the UK National Institute for Health and Care Excellence (NICE) guidelines which uses clinical factors alone [48]. Many trials in this review may have benefited from using prediction models, which utilise a combination of specific variables and may offer higher predictive ability [48]. With no study in this review using a validated prediction model to identify high-risk women and a lack of externally validated prediction algorithms, future studies assessing the effect of micronutrient supplementation interventions in women at high risk of developing pre-eclampsia could use validated prediction models to select participants, with more trials also exploring alternative combinations of predictive factors that accurately determine women who are at high risk of developing pre-eclampsia.

The trials included in this review also varied in other aspects of study design and reporting of findings. In addition to differences in the identification of high-risk women, there was variability in the eligibility criteria, with one trial including women with other co-morbidities, for example, kidney disease [38]. Furthermore, some trials reported the diagnostic criteria of severe pre-eclampsia, but several did not [31, 33, 36, 39, 41] while other trials did not report a diagnostic criterion for any classification of pre-eclampsia [29, 31, 32, 39]. There was a lack of reporting on statistical methodology, in particular the inclusion of confounding variables in the analysis. Several trials did not clearly state whether confounding variables had been adjusted for. In those that did, the most common confounding factors adjusted for were maternal age and BMI. Compliance across the trials ranged from moderate to high, with the majority of studies using tablet count while Zheng et al. used plasma homocysteine levels as a method of demonstrating the potential confounding effect of compliance on the outcome of pre-eclampsia. Data for compliance was not reported in several trials [23, 26–28, 30, 31, 38, 39] while there was also variation across trials in the frequency, dosing and timing of supplementation, while the gestational age at the time of intervention was often not reported.

Two trials [18, 32] commenced in the preconception period. Calcium is known to have a benefit in reducing the risk of pre-eclampsia from 20 weeks’ gestation onwards, however our findings suggest that calcium supplementation before this point may not necessarily be effective as is shown by Hofmeyr et al. [22]. On the other hand, Zheng *et al*. reported that supplementation of folic acid from preconception to delivery may effectively reduce risk of severe pre-eclampsia [36]. Micronutrient supplementation interventions may also be effective for different durations and at different time points, which could be investigated in future trials to further elucidate the effect of these micronutrients. However, caution is imperative as our findings show that some micronutrient interventions, notably vitamin C and E, are not entirely benign.

### Strengths and limitations

This systematic review has several strengths. This study addressed whether micronutrient interventions are effective in reducing the development of pre-eclampsia in women identified as high risk for the condition. Previous reviews have focused on an unselected approach, often with no risk stratification. The identification of women with an increased risk of developing pre-eclampsia might enable targeted intervention in women most likely to benefit. This work complements previous findings that calcium and vitamin D are beneficial in reducing pre-eclampsia including in those identified as high risk, whilst highlighting the need for more large-scale well-powered studies with improved and more consistent reporting of interventions and findings. A comprehensive search strategy was used to screen for studies that targeted interventions at higher risk populations by using a pre-specified eligibility criteria. The findings from this review are important to inform the design of future RCTs to improve the data quality and clarify the effects of micronutrient supplementation, particularly in women at high risk for pre-eclampsia and the effect on different classifications of pre-eclampsia.

This review has limitations. It only included studies published in English which may have introduced publication bias. The high risk of bias in several studies in this review highlights the low-quality evidence in this research area and supports the need for more robust future trials. Additionally, many trials with significant results were not adequately powered to detect a difference in pre-eclampsia between treatment groups or may have overestimated the effect of the given intervention as a result of a small sample size. The analysis was limited in scope as pooling of data were not possible for all micronutrient interventions, in addition to a lack of adjustment for potential confounders. Furthermore, there was high methodological variability between studies particularly with calcium, vitamin D and vitamin C and E trials, limiting the consistency of data across these studies. One reason for this may be because of differences in the interventions themselves, as Herrera et al. did not investigate calcium alone but in combination with linoleic acid, whilst Samimi et al. investigated vitamin D given with calcium. Additionally, the gestational age at which interventions were administered varied significantly amongst trials. Finally, the risk factors used to screen for women at high risk of pre-eclampsia differed significantly between trials.

### Recommendations for further research and practice

Our review highlighted the lack of studies of interventions targeting higher risk women in the preconception period. Only two studies [22, 36] in this review initiated the intervention in the preconception period. With symptoms of pre-eclampsia beginning from 20 weeks’ gestation, preconception and early pregnancy interventions require further exploration as they may provide greater insight into how to improve maternal and neonatal outcomes. We have reported a lower rate of pre-eclampsia with calcium and vitamin D, however, conclusions were limited by small sample sizes, methodological variability and heterogeneity between studies. Future studies of these two micronutrients are warranted, however they must be larger-scale and well-powered to allow more thorough and reliable conclusions to be drawn. Additionally, several studies [24, 25, 30, 38, 39] in this review did not clearly state the gestation of the participants at the beginning and end of the given micronutrient supplementation intervention, as well as failing to report on adherence and compliance. Better reporting of trials is required in future studies to ascertain the relationship between the effectiveness of the given micronutrient supplementation intervention and the timing with which it is administered. Further studies using externally validated prediction models that have demonstrated higher predictive performance such as models by Poon et al. [49] and Odibo et al. [50] with a detection rate of 91.7% and 80.0% respectively for pre-eclampsia requiring early delivery and use a variety of predictive factors such as chronic hypertension and PAPP-A may additionally clarify the effects of these micronutrients. Finally, future research is needed to determine the effect of micronutrient supplementation interventions on different classifications of pre-eclampsia, from mild to superimposed pre-eclampsia, to progress towards a more personalised and tailored approach in the primary prevention of pre-eclampsia.

## Conclusion

This study showed a small effect of calcium and vitamin D in the prevention of pre-eclampsia in women who were identified as higher risk of developing the condition. The review was limited by the inclusion of studies with a small sample size. Significant heterogeneity between studies as well as methodological variability was evident. Further higher quality, large-scale RCTs of calcium and vitamin D, the use of prediction modelling, and particularly at different points of time before and during pregnancy, may be beneficial to assess the efficacy of micronutrient supplementation intervention in the prevention of pre-eclampsia in high-risk women.

## Supplementary information

Supplementary Information
