# Supplementary material for: Micronutrient supplementation interventions in preconception and pregnant women at increased risk of developing pre-eclampsia: a systematic review and meta-analysis
Source: Eur J Clin Nutr. 2022 Nov 9;77(7):710–30. doi: 10.1038/s41430-022-01232-0 (PMC10335932; doi:10.1038/s41430-022-01232-0)
Supplement: Supplementary file 1 — Supplementary Information [file 41430_2022_1232_MOESM1_ESM.docx]

The supplementary information includes the search strategies and risk of bias summary.

**Supplementary Information 1. Search strategies used for MEDLINE, EMBASE and the Cochrane Central Register of Controlled Trials**

MEDLINE

| 1 | Pregnancy/ |
| --- | --- |
| 2 | pregnan*.mp. |
| 3 | gravid*.mp. |
| 4 | gestation*.mp. |
| 5 | Pregnant Women/ |
| 6 | prepregnan*.mp. |
| 7 | pre-pregnan*.mp. |
| 8 | preconception*.mp. |
| 9 | (child adj3 bearing).mp. |
| 10 | (child* adj3 bear*).mp. |
| 11 | matern*.mp. |
| 12 | prenatal.mp. |
| 13 | pre-natal.mp. |
| 14 | perinatal.mp. |
| 15 | peri-natal.mp. |
| 16 | antenatal.mp. |
| 17 | ante-natal.mp. |
| 18 | or/1-17 |
| 19 | exp Vitamins/ |
| 20 | vitamin*.mp. |
| 21 | exp Minerals/ |
| 22 | mineral*.mp. |
| 23 | exp Micronutrients/ |
| 24 | micronutrient*.mp. |
| 25 | micro-nutrient*.mp. |
| 26 | exp Diet/ |
| 27 | diet*.mp. |
| 28 | nutri*.mp. |
| 29 | exp Dietary supplements/ |
| 30 | supplement*.mp. |
| 31 | or/19-30 |
| 32 | exp Pre-Eclampsia/ |
| 33 | pre-eclampsia.mp. |
| 34 | preeclampsia.mp. |
| 35 | exp Eclampsia/ |
| 36 | eclampsia.mp. |
| 37 | ((pre-eclamp$ or preeclamp$) or (pre adj3 eclamp$)).mp. |
| 38 | toxaemia.mp. |
| 39 | toxemia.mp. |
| 40 | ((toxemi$ or toxaemi$) adj3 pregnan$).mp. |
| 41 | exp Hypertension, Pregnancy-Induced/ |
| 42 | gestational hypertension.mp. |
| 43 | hypertens*.mp. |
| 44 | (hyperten$ adj3 pregnan$).mp. |
| 45 | exp Pregnancy Complications/ |
| 46 | pregnancy complication*.mp. |
| 47 | (complication* adj3 pregnan$).mp. |
| 48 | or/32-47 |
| 49 | "randomized controlled trial".pt. |
| 50 | "controlled clinical trial".pt. |
| 51 | (random$ or placebo$).tw,sh. |
| 52 | ((singl$ or double$ or triple$ or treble$) and (blind$ or mask$)).tw,sh. |
| 53 | single-blind method/ |
| 54 | double-blind method/ |
| 55 | or/49-54 |
| 56 | 18 and 31 and 48 and 55 |
| 57 | (animals not human).mp. |
| 58 | 56 not 57 |

EMBASE

| 1 | Pregnancy/ |
| --- | --- |
| 2 | pregnan*.mp. |
| 3 | gravid*.mp. |
| 4 | gestation*.mp. |
| 5 | Pregnant Women/ |
| 6 | prepregnan*.mp. |
| 7 | pre-pregnan*.mp. |
| 8 | preconception*.mp. |
| 9 | (child adj3 bearing).mp. |
| 10 | (child* adj3 bear*).mp. |
| 11 | matern*.mp. |
| 12 | prenatal.mp. |
| 13 | pre-natal.mp. |
| 14 | perinatal.mp. |
| 15 | peri-natal.mp. |
| 16 | antenatal.mp. |
| 17 | ante-natal.mp. |
| 18 | or/1-17 |
| 19 | exp Vitamins/ |
| 20 | vitamin*.mp. |
| 21 | exp Minerals/ |
| 22 | mineral*.mp. |
| 23 | exp Micronutrients/ |
| 24 | micronutrient*.mp. |
| 25 | micro-nutrient*.mp. |
| 26 | exp Diet/ |
| 27 | diet*.mp. |
| 28 | nutri*.mp. |
| 29 | exp Dietary supplements/ |
| 30 | supplement*.mp. |
| 31 | or/19-30 |
| 32 | exp Pre-Eclampsia/ |
| 33 | pre-eclampsia.mp. |
| 34 | preeclampsia.mp. |
| 35 | exp Eclampsia/ |
| 36 | eclampsia.mp. |
| 37 | ((pre-eclamp$ or preeclamp$) or (pre adj3 eclamp$)).mp. |
| 38 | toxaemia.mp. |
| 39 | toxemia.mp. |
| 40 | ((toxemi$ or toxaemi$) adj3 pregnan$).mp. |
| 41 | exp Hypertension, Pregnancy-Induced/ |
| 42 | gestational hypertension.mp. |
| 43 | hypertens*.mp. |
| 44 | (hyperten$ adj3 pregnan$).mp. |
| 45 | exp Pregnancy Complications/ |
| 46 | pregnancy complication*.mp. |
| 47 | (complication* adj3 pregnan$).mp. |
| 48 | or/32-47 |
| 49 | Clinical trial/ |
| 50 | randomized controlled trial/ |
| 51 | controlled clinical trial/ |
| 52 | multicenter study/ |
| 53 | Phase 3 clinical trial/ |
| 54 | Phase 4 clinical trial/ |
| 55 | exp RANDOMIZATION |
| 56 | Single Blind Procedure/ |
| 57 | Double Blind Procedure/ |
| 58 | Crossover Procedure/ |
| 59 | PLACEBO/ |
| 60 | randomi?ed controlled trial$.tw. |
| 61 | rct.tw. |
| 62 | (random$ adj2 allocat$).tw. |
| 63 | single blind$.tw. |
| 64 | double blind$.tw. |
| 65 | ((treble or triple) adj blind$).tw. |
| 66 | placebo$.tw. |
| 67 | Prospective Study/ |
| 68 | or/49-67 |
| 69 | 18 and 31 and 48 and 68 |
| 70 | (animals not human).mp. |
| 71 | 69 not 70 |

Cochrane Central Register of Controlled Trials

| 1 | Pregnan* |
| --- | --- |
| 2 | MeSH descriptor: [Pregnancy] explode all trees |
| 3 | Gestation |
| 4 | Pregnant women |
| 5 | MeSH descriptor: [Pregnant Women] explode all trees |
| 6 | Expect* |
| 7 | mother OR matern* |
| 8 | gravidity |
| 9 | MeSH descriptor: [Gravidity] explode all trees |
| 10 | Prepregnan* OR preconception OR periconception |
| 11 | Antenatal* OR Prenatal* OR Perinatal* |
| 12 | #1 OR #2 OR #3 OR #4 OR #5 OR #6 OR #7 OR #8 OR #9 OR #10 OR #11 |
| 13 | Pre-eclampsia |
| 14 | MeSH descriptor: [Pre-Eclampsia] explode all trees |
| 15 | Gestational Hypertension |
| 16 | MeSH descriptor: [Hypertension, Pregnancy-Induced] explode all trees |
| 17 | MeSH descriptor: [Hypertension] explode all trees and with qualifier(s): [prevention & control – PC] |
| 18 | MeSH descriptor: [Pregnancy Complications, Cardiovascular] explode all trees and with qualifier(s): [prevention & control – PC] |
| 19 | #13 OR #14 OR #15 OR #16 OR #17 OR #18 |
| 20 | Vitamins |
| 21 | MeSH descriptor: [Vitamins] explode all trees |
| 22 | Micronutrients |
| 23 | MeSH descriptor: [Micronutrients] explode all trees |
| 24 | Minterals |
| 25 | MeSH descriptor: [Minerals] explode all trees |
| 26 | Dietary supplements |
| 27 | MeSH descriptor: [Dietary Supplements] explode all trees |
| 28 | Supplement* |
| 29 | Multivitamins |
| 30 | #20 OR #21 OR #22 OR #23 OR #24 OR #25 OR #26 OR #27 OR #28 OR #29 |
| 31 | MeSH descriptor: [Animals] explode all trees |
| 32 | MeSH descriptor: [Humans] explode all trees |
| 33 | #31 NOT #32 |
| 34 | #12 AND #19 AND #30 |
| 35 | #34 NOT #33 |

**Supplementary Information 2. ‘Risk of bias’ summary: authors’ judgements about each risk of bias item for each included study**

| Quality assessment criteria | | | | | | | |
| --- | --- | --- | --- | --- | --- | --- | --- |
| Reference | A | B | C | D | E | F | Overall Bias |
| Hofmeyr et al. 2019 |  |  |  |  |  |  |  |
| Baba Dizavandy et al. 1998 |  |  |  |  |  |  |  |
| Behjat et al. 2017 |  |  |  |  |  |  |  |
| Chappell et al. 1999 |  |  |  |  |  |  |  |
| De Araujo et al. 2020 |  |  |  |  |  |  |  |
| Herrera et al. 1998 |  |  |  |  |  |  |  |
| Kalpdev et al. 2010 |  |  |  |  |  |  |  |
| Karamali et al. 2015 |  |  |  |  |  |  |  |
| Niromanesh et al. 2001 |  |  |  |  |  |  |  |
| Parrish et al. 2013 |  |  |  |  |  |  |  |
| Samimi et al. 2015 |  |  |  |  |  |  |  |
| Sanchez-Ramos et al. 1994 |  |  |  |  |  |  |  |
| Spinatto et al. 2007 |  |  |  |  |  |  |  |
| Vadillo-Ortega et al. 2011 |  |  |  |  |  |  |  |
| Zheng et al. 2020 |  |  |  |  |  |  |  |
| Villar et al. 2009 |  |  |  |  |  |  |  |
| Azami et al. 2017 |  |  |  |  |  |  |  |
| Beazley et al. 2005 |  |  |  |  |  |  |  |
| Poston et al. 2006 |  |  |  |  |  |  |  |
| Wen et al. 2018 |  |  |  |  |  |  |  |

Risk of bias legend

(A) Bias arising from the randomization process - Selection bias

(B) Bias due to deviations from intended interventions (assignment)

(C) Bias due to deviations from intended interventions (adherence)

(D) Bias due to missing outcome data - Attrition bias

(E) Bias in measurement of the outcome - Measurement bias

(F) Bias in selection of the reported result - Reporting bias

Low risk of bias

Some concerns

High risk of bias
